# CAN NOVICE LEARNERS BE CLINICAL CHANGE AGENTS FOR DEMENTIA CARE PRACTICE?

**DOI:** 10.1093/geroni/igae098.3107

**Published:** 2024-12-31

**Authors:** Edmund Duthie, Kathryn Denson, Deborah Simpson, Stacy Barnes, Wendy Betley, Amanda Szymkowski, Steven Denson

**Affiliations:** Medical College of Wisconsin, Milwaukee, Wisconsin, United States; Medical College of Wisconsin, Milwaukee, Wisconsin, United States; University of Wisconsin School of Medicine and Public HealthH, Milwaukee, Wisconsin, United States; Marquette University College of Nursing, Milwaukee, Wisconsin, United States; Alzheimer’s Association, Chicago, Illinois, United States; Medical College of Wisconsin, Milwaukee, Wisconsin, United States; Medical College of Wisconsin, Milwaukee, Wisconsin, United States

## Abstract

**Intro:**

Caregiver support is an essential component in dementia care and yet clinicians’ referrals to community-based support services are lacking. Direct Connect (DC) is a clinician-initiated referral process for dementia patients and caregivers, offered by the Alzheimer Association to make the process simple and efficient.

**Methods:**

We created a brief educational intervention targeting medical, nursing, and pharmacy students to develop them into DC champions at clinical rotation sites in Wisconsin. Content focused on dementia detection, caregiver burden, available resources, and DC referrals. Delivery included online, in-person, and hybrid formats. Data was collected with a post-intervention survey and student interviews. At baseline (2021), 277 DC referrals were made by clinicians in Wisconsin.

**Results:**

Following a 2-year rollout to students (N=375), the annual number of DC referrals received from clinicians increased (300 in 2022; 351 in 2023). However, review of referral data revealed the increase was primarily from licensed clinicians not at student champion sites. Follow-up interviews with students indicated they find it daunting to suggest changes in busy clinical settings where they lack experience and autonomy. Despite incorporating scripting and role playing into our educational session, students continued to lack confidence.

**Discussion:**

Although student champions have proven effective in other clinical conditions, our results suggest different strategies are needed for dementia care. Based on these findings, we propose to refocus training efforts toward more senior learners such as medical residents who have more experience and clinical autonomy, and to embed the DC referral within the electronic health record to facilitate usage.

